# Assessment of the Herd Management Effects on Mastitis Frequency in Austrian Dairy Farms

**DOI:** 10.1111/asj.70155

**Published:** 2026-01-26

**Authors:** Muhammed Mert Sertkaya, Christa Egger‐Danner, Thomas Wittek

**Affiliations:** ^1^ Clinical Centre for Ruminant and Camelid Medicine University of Veterinary Medicine Vienna (Vetmeduni) Vienna Austria

**Keywords:** cattle, management, mastitis, SCC, udder health

## Abstract

Mastitis, an inflammation of mammary tissue caused by infection, physical injury, or chemical irritation, is one of the most economically significant diseases in the global dairy industry. Both acute and chronic forms of mastitis lead to an increase in somatic cell counts (SCCs) in milk and a significantly reduced milk yield. Farmers' knowledge and awareness of mastitis play a crucial role for the preventive and therapeutic measures to manage the disease. This study aims to assess the efficacy of mastitis control practices adopted by dairy farmers in Austria by a survey‐based approach. Data on milk yield and milk components were obtained from the Austrian National Milk Recording System, whereas farm management practices were documented by direct surveys conducted with dairy farmers. The study found that farm management practices related to milking, udder health, hygiene management, disease control, and nutrition significantly impact milk yield and the frequency of SCC exceeding 200,000/mL. These findings highlight the critical influence of management strategies during the dry period, nutrition, and milking practices on both milk production and mastitis indicators. Based on these results, it is strongly recommended that farmers receive training on effective strategies to improve milk yield and control mastitis.

## 1 Introduction

Mastitis, the inflammation of mammary tissue due to infection, physical injury, or chemical irritation, is one of the most economically important diseases in the dairy industry all over the world (Ashraf and Imran 2020; Goulart and Mellata 2022; Tommasoni et al. 2023). Both acute and chronic forms of mastitis increase SCC in milk, particularly through an influx of neutrophils (primary leukocytes) into the mammary glands and milk (Rinaldi et al. 2010). Numerous studies indicate how mastitis influences milk composition, notably through increased SCC, which correlates with decreased concentrations of casein, lactose, and milk fat, whereas enzymatic activities increase (Ballou et al. 1995; Braunschweig et al. 2000). These alterations affect milk quality and yield (Malek dos Reis et al. 2013). Cows with increased SCC have a significantly decreased milk yield (Koldeweij et al. 1999). First‐lactation cows experience a 3%–9% daily milk yield loss, and multiparous cows face a 4%–18% loss when SCCs reach 500,000/mL (Hagnestam‐Nielsen et al. 2009). However, elevated SCC is not just an indicator of abnormal conditions in udder tissue; high SCCs are also associated with lower milk quality, reduced cheese yield, and a shorter shelf life for milk (Volpe et al. 2016). Elevated SCC serves as a marker for udder health and results in lower milk prices due to quality deficits. Reports in the United States estimated that elevated SCC in bulk tank milk causes an annual economic loss of roughly $810 million, whereas in the United Kingdom, bovine mastitis accounts for annual losses ranging from £57 million to £185 million (Volpe et al. 2016).

Most mastitis research has focused on epidemiology and control (Barlow 2011). Antibiotics are commonly used to control mastitis. However, their overuse, especially for subclinical cases, is contributing to the development of antimicrobial resistance. Thus, it is essential to encourage dairy farmers to use antibiotics responsibly (Barlow 2011). Permanent emphasis on awareness and education about responsible antibiotic application is crucial for controlling the disease without exacerbating resistance against the antimicrobial drugs (Sharun et al. 2021). Farmers' knowledge and awareness of mastitis significantly influence the preventive and treatment measures they adopt for managing and controlling the disease (Abraham and Zeleke 2017). Lack of awareness about mastitis, combined with limited financial resources, can prevent or delay the adoption of practices aimed at producing high‐quality milk (Nimbalkar et al. 2020). Studies indicate that improving farmers' understanding of mastitis is associated with a decrease in mastitis frequency and a notable increase in economic returns (Lam et al. 2013). Increasing education and awareness among farmers is therefore essential to promoting effective mastitis management practices that can enhance both herd health and profitability.

With an average herd size of approximately 20 dairy cows, Austrian dairy farms have relatively small herds compared to other European countries (Firth et al. 2019). However, the number of farms with more than 50 cows increased tenfold from 137 farms in 2000 to 1366 in 2017. As herd sizes grew, large herds accounted for a quarter of the nation's milk production (Egger‐Danner et al. 2020). In 2006, a health monitoring system was implemented in Austria to record health data for dairy herds, including SCC and different mastitis indicators to monitor mastitis prevalence (Egger‐Danner et al. 2012). According to available data, over 60% of Austrian dairy farms report mastitis (Egger‐Danner 2025, personal communication).

General herd management practices have a significant impact on udder health (Belage et al. 2017). In the literature, numerous studies have reported associations between management strategies implemented on dairy farms and various udder health indicators (Dufour et al. 2011). Several management practices have been consistently linked to herd‐level SCC when applied under typical dairy farm conditions (Dufour et al. 2011). The dry period is particularly important in dairy cows, as both persistent infections from the previous lactation and new intramammary infections acquired during this time can contribute to the development of subclinical and clinical mastitis in the subsequent lactation, ultimately reducing milk yield (Gundelach et al. 2011). The primary goal of dry period udder health management is to minimize the prevalence of intramammary infections (IMI) at calving (Dufour et al. 2019).

The study aims to evaluate the efficacy of the husbandry and management practices implemented by dairy farmers in Austria to control mastitis by a survey study. If various farm management strategies influence udder health and mastitis prevalence, then bacterial culturing before dry‐off treatment can serve as a key prognostic indicator for udder health postcalving. Additionally, the selective application of medicinal dry‐off products, such as antibiotics and teat sealants, may also play a crucial role in predicting udder health outcomes.

It was hypothesized that differences in milking practice (e.g., milking frequency and machine maintenance) have an effect on milk yield and that the frequency of disinfection of milking equipment is negatively associated with mastitis frequency. A second hypothesis was that improved hygiene standards applied on farms decrease the frequency of mastitis cases.

## 2 Materials and Methods

### 2.1 Data

Data on milk yield and milk components were sourced from the Austrian National Milk Recording System (LKV), whereas information about the dairy farms included in the research was collected directly from dairy farmers using a questionnaire. All farmers signed a written consent to take part in the study and were allowed to use their data. The collected data covers the period from October 1, 2020, to September 30, 2021, and was gathered within the D4Dairy project (https://d4dairy.com/en/). Average cow numbers per farm were calculated by dividing the total days cows that were present on each farm by the collection period's duration.

Questionnaire (Supporting Information S1): Information was gathered from farmers on various aspects of farm management: milking practices, udder health, hygiene management, disease control, and nutrition. Specific aspects were milking duration, premilking routines, milking hygiene, milking machine features (e.g., automatic cluster removal and shut‐off), on‐farm disinfection, postmilking procedures, dry period management, ketosis prevention, and herd, udder, and bedding hygiene. Some survey questions were structured as open‐ended or optional to encourage detailed responses from farmers.

The survey data were recorded within the D4Dairy project, where an announcement was made. Mastitis was evaluated based on SCC measurements, with thresholds set at SCC of 200,000/mL as an indicator of subclinical mastitis and cows exceeding SCC three times above 200,000/mL as a marker of recurring and/or chronic mastitis (Egger‐Danner et al. 2020). Additional data from farms included the number of cows with at least one initial diagnosis of udder health issues within the study period.

### 2.2 Statistical Analysis

Quantitative data, such as the number of milking cows present in dairy farms, were recorded by computing the averages based on the period of the research survey. The average number of cows was therefore calculated by dividing the number of days that cows were present at the farm from the reference period by 365. Data are displayed as standard deviation, median, minimum and maximum, percentages, and numbers. Normality of the continuous variables was assessed using the Shapiro–Wilk test, Kolmogorov–Smirnov test, Q‐Q plots, and the measures of skewness and kurtosis.

Comparisons involving two independent groups were carried out using the independent samples *t* test if the assumption of normality was met; otherwise, the Mann–Whitney *U* test was applied. For comparisons involving more than two independent groups, ANOVA was used when the data were normally distributed. Tukey's post hoc analysis was conducted when the assumption of homogeneity of variances was satisfied, whereas Tamhane's *T*2 test was used for unequal variances. If the assumption of normality was violated, the Kruskal–Wallis test was applied. In such cases, post hoc analyses were performed using appropriate nonparametric methods such as Dunn's test with Bonferroni correction.

For investigating associations between two continuous variables, Pearson's correlation was applied if data were normally distributed; Spearman's correlation was used otherwise. ROC analysis was performed for questions addressing specific farm practices such as “Is a milking order maintained (with cows with higher cell counts, treated cows, or cows known to have chronic udder problems milked last)?”, “Does every cow have an area where to feed immediately postmilking?”, and “Have there been any changes in the drying‐off practice in the last 10 years (drying‐off procedure, BU, or use of teat sealants or antibiotic drying agents)?” undefined, showed a nonuniform distribution.

All statistical analyses were performed by using IBM SPSS Statistics, Version 20 (IBM Corp., Armonk, NY, United States). The significance level was set at *p* < 0.05.

## 3 Results

The data on udder health and milking practices in herd management were collected from October 1, 2020, to September 30, 2022, on 411 Austrian dairy farms. These variables included the mean number of cows per farm, milk production, SCC, percentage of milk records over SCC above 200,000/mL within the observation period, the percentage of cows recording an SCC above 200,000/mL on three or more occasions, and the percentage of cows with at least one initial diagnosis of udder health issues. These are summarized in Table 1 below. The statistical analysis results for the questions asked in the questionnaire are given in Supporting Information S2.

**TABLE 1:** Data on the number of cows, the amount of milk, and so on in 411 Austrian dairy farms during the research period.

Parameter	Arithmetic mean	Standard deviation	Minimum	Maximum	CI (95.0%)
*nCow*	45.76	20.52	15.26	140.5	43.78–47.74
Milk yield (kg)	8921.18	1372.78	3690.95	13,871.89	8788.50 – 9053.90
SCC (× 10^3^/mL)	177.89	76.98	51.81	431.80	170.45–185.33
Percentage of milk records in which the SCC in the herd exceeded 200,000 during the relevant observation period	17.94	8.18	2.94	48.55	17.15–18.73
Percentage of cows exceeding three times the SCC 200,000/mL during the observation period	17.03	9.78	0	50.59	16.08–17.98
Percentage of cows with at least one initial diagnosis of udder health issues	10.62	9.54	0	47.54	9.70–11.54

**FIGURE 1:**
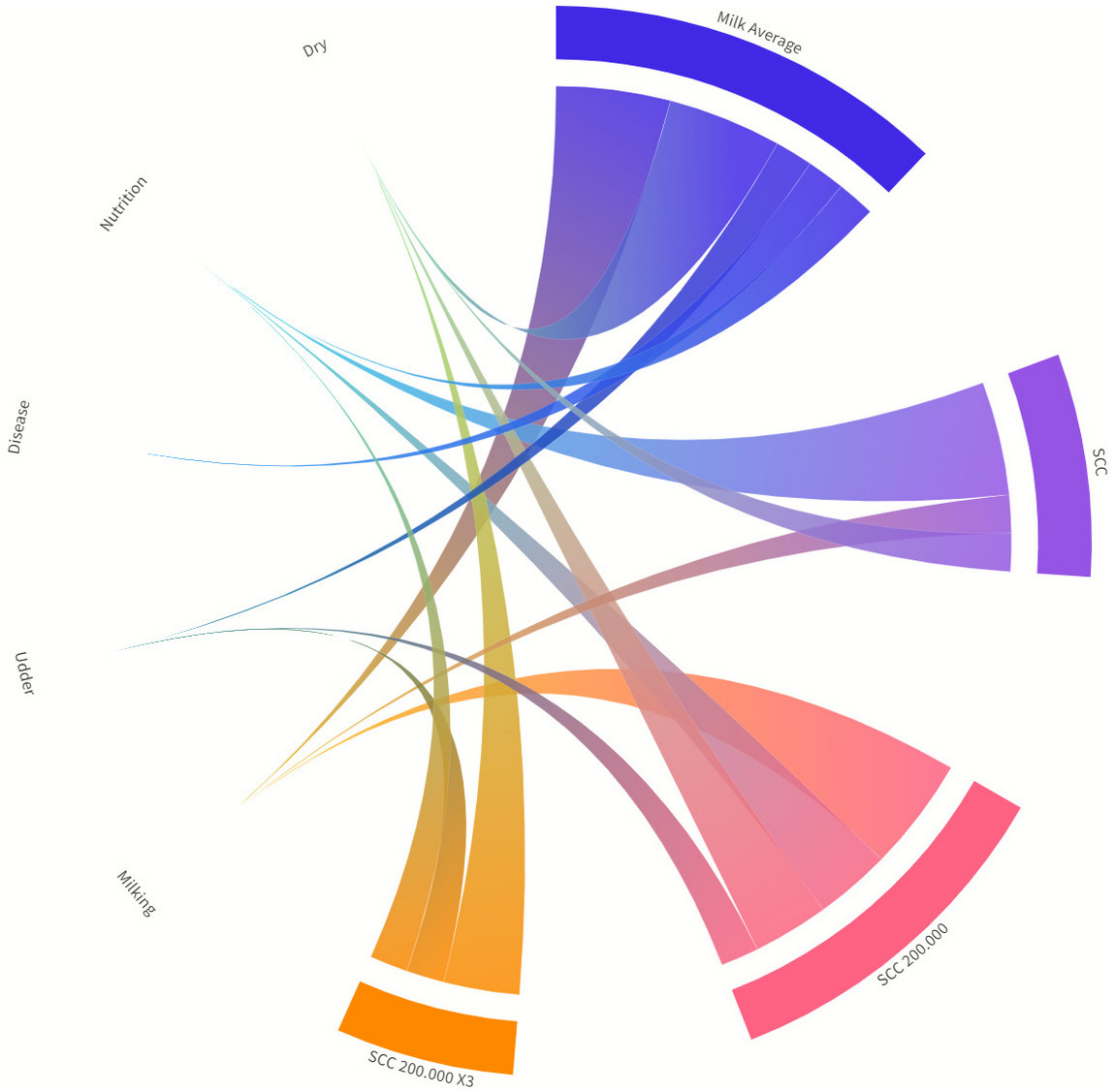
Chord diagram showing relationships among variables, including the mean milk production, SCC, the percentage of milk records where herd SCC > 200,000/mL, the percentage of cows with SCC > 200,000/mL three times or more and other variables comprising milking practices, udder health, hygiene management, disease control, and nutrition practices. SCC 200,000: Percentage of cows exceeding three times 200,000 somatic cell count during the observation period.

In this study, a correlation analysis was conducted among variables, including average milk yield, SCC, the percentage of milk records with SCC exceeding 200,000/mL, the percentage of cows surpassing the 200,000/mL threshold on three or more occasions, and how many animals have hock injuries or swelling, along with other numerical data collected from farmers (Table 2). Figure 1 illustrates the relationships between herd management practices and udder health indicators. Average milk yield was most strongly associated with milking practices and dry period management. SCC was mainly linked to nutrition practices, as well as to milking practices and dry period management. The occurrence of SCC > 200,000 was most strongly related to milking practices but also showed weaker associations with dry period management, nutrition practices, and udder health. In contrast, repeated high SCC values (SCC > 200,000 × 3) were predominantly associated with dry period management. These findings suggest that different management practices contribute to milk yield and udder health outcomes through distinct yet interconnected pathways.

**TABLE 2:** Correlation analysis between variables including average milk yield, SCC, percentage of milk records with SCC > 200,000/mL, percentage of cows with SCC > 200,000/mL at least in three times, average time spent in milking activities, time spend for milking, the number of animals with hock injuries or swelling, and animal classification based on udder cleanliness levels.

Correlations		Milk yield	SCC	Percentage of milk records in which the SCC exceeded 200,000/mL during the relevant observation period	Percentage of cows exceeding 3 times the SCC of 200,000/mL during the observation period	How long is the average duration of the milking work per milking (time in minutes)?	How many animals have hock injuries or swelling?	Grade 1 udder cleanliness^a^	Grade 2 udder cleanliness^a^	Grade 3 udder cleanliness^a^	Grade 4 udder cleanliness^a^
Milk yield	*r*	1.000	0.036	−0.018	−0.038	0.187	0.075	0.119	−0.091	−0.158	−0.108
	*p*		0.465	0.718	0.447	0.001	0.151	0.017	0.073	0.006	0.110
	*N*	411	411	411	411	295	369	402	391	302	221
SCC	*r*		1.000	0.917	0.843	0.040	−0.016	0.087	−0.089	−0.031	−0.024
	*p*			0.000	0.000	0.493	0.766	0.081	0.080	0.595	0.724
	*N*		411	411	411	295	369	402	391	302	221
Percentage of milk records in which the SCC exceeded 200,000/mL during the observation period	*r*			1.000	0.932	0.030	−0.029	0.094	−0.094	−0.020	−0.027
	*p*				0.000	0.613	0.574	0.060	0.064	0.731	0.693
	*N*			411	411	295	369	402	391	302	221
Percentage of cows exceeding three times the SCC of 200,000/mL during the observation period	*r*				1.000	0.026	−0.025	0.093	−0.101	−0.043	−0.032
	*p*					0.653	0.627	0.063	0.046	0.454	0.639
	*N*				411	295	369	402	391	302	221
How long is the average duration of the milking work per milking (time in minutes)?	*r*					1.000	0.050	0.008	0.002	−0.054	−0.070
	*p*						0.419	0.887	0.970	0.434	0.389
	*N*					295	268	288	282	211	154
How many animals have hock injuries or swelling?	*r*						1.000	−0.277	0.183	0.284	0.243
	*p*							0.000	0.001	0.000	0.000
	*N*						369	363	353	276	207

^a^ Grade 1 udder cleanliness: Free of dirt; Grade 2 udder cleanliness: Slightly dirty (2%–10% of the surface area); Grade 3 udder cleanliness: Moderately covered with dirt (10%–30% of the surface area); Grade 4 udder cleanliness: Heavily soiled, with caked dirt covering more than 30% of the surface area (Cook and Reinemann 2007).

All received answers about the methods and techniques used by farmers in the milking process were addressed under the category of milking methods. Information received on questions such as cleanliness of the teat and scoring of contamination in the udder quarters was addressed under the category of udder health. Information received on issues such as disinfection and litter within the farm was addressed under the hygiene management category. Information obtained on diseases such as ketosis was evaluated under the category of disease control, information obtained on the dry period was evaluated under the category of dry period, and freshness of the feed and nutrition after milking were evaluated under the category of nutrition. Figure 1 presents the distribution of responses from Austrian dairy farmers to questions regarding milking methods, udder health, hygiene management, disease control, and nutrition practices.

**FIGURE 2:**
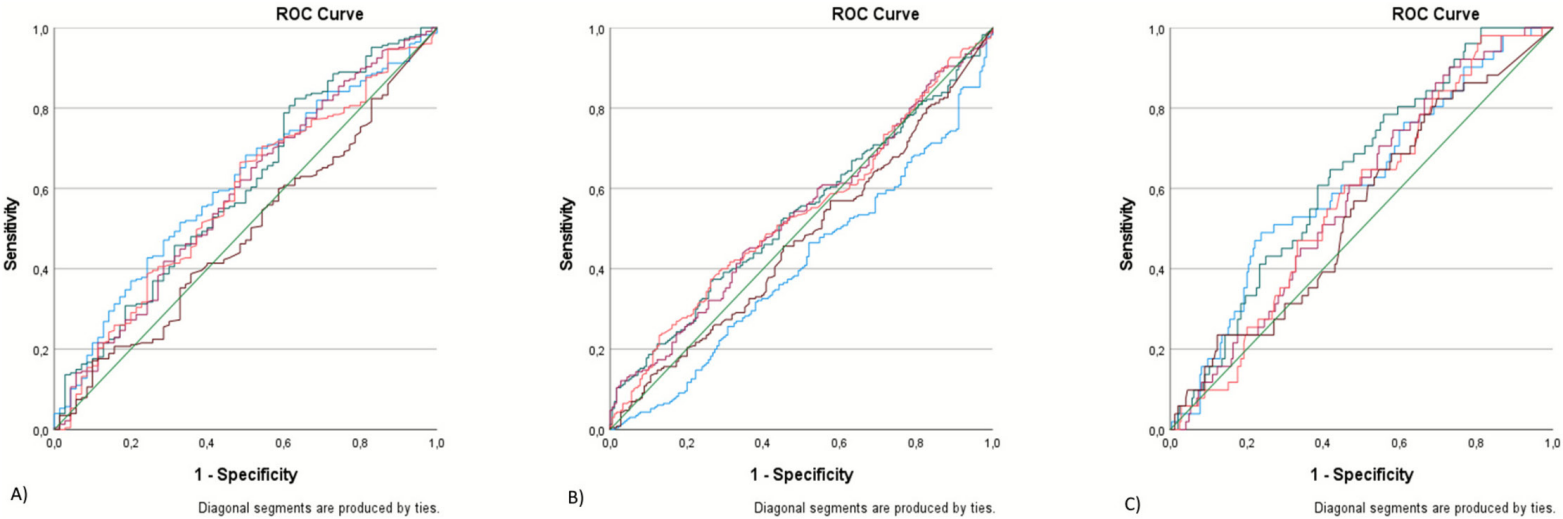
ROC analysis to examine the relation between the following questions: “Is a milking order maintained (cows with increased SCC, treated cows or cows known to have chronic udder diseases are milked last)?”, “Is there a feeding place for each cow after milking?”, and “Has there been any change in drying in the last 10 years?” regarding following variables: average milk production, SCC, percentage of herd milk records with SCC > 200,000/mL, percentage of cows exceeding an SCC of 200,000/mL more than three times, and the percentage of cows with at least one initial diagnosis of udder health issues. (A) Is a milking order maintained (where cows with increased cell counts, treated cows, or cows with chronic udder diseases are milked last)? (B) Has there been any change in drying in the last 10 years? (C) Is there a feeding place for each cow after milking? It is seen that milk average is effective with all three management models. Lines: blue: milk average; green: SCC, pink: percentage of herd milk record with SCC > 200,000/mL, red: percentage of cows exceeding a SCC of 200,000/mL more than three times, brown: percentage of cows with at least one initial diagnosis of udder health problems.

The statistical significance of the results for data that were not homogeneously distributed was evaluated using ROC analysis (Figure 2). In particular, this analysis was applied to responses regarding whether a milking order is maintained (i.e., whether cows with increased SCC, treated cows, or cows with chronic udder diseases are milked last), whether each cow has a feeding place after milking, and whether any changes in drying practices have occurred in the last 10 years. The ROC analysis enabled the assessment of the discriminatory capacity of these management practices with respect to udder health indicators.

In this study, a positive correlation was observed between average milk yield and milking time, as well as udder cleanliness graded at Level 1 (Table 2). Conversely, a negative correlation was identified with udder cleanliness graded at Level 3. Udder cleanliness was significantly associated with average milk yield, with farms implementing udder cleaning procedures, regardless of the specific method, demonstrating statistically higher yields than those without cleaning (*p* < 0.05). Additionally, farms lacking an automatic cluster removal system, or those not using it consistently, showed a lower average milk yield (*p* < 0.05), possibly due to prolonged milking times and increased teat‐end stress associated with delayed cluster removal.

Milk yields were also influenced by milking order (Table 3). Farms that had a structured milking order realized higher yields than farms milking cows in a random order (*p* < 0.05). Specifically, milking cows with high SCC, treated cows, or those suffering from chronic mastitis after healthy cows positively impacted yield (*p* < 0.05). Also, having a designated feeding space for each cow postmilking contributed to increased milk yield (*p* < 0.05), whereas farms that did not apply teat dipping after milking reported less yield performance of milk production (*p* < 0.05).

**TABLE 3:** Herd management practices found to be statistically significant with milk records having SCC > 200,000/mL during the observation period.

	Percentage of milk records in which the SCC > 200,000/mL during the observation period							
Question	Category	Valid *N*	Mean	Standard Deviation	Median	Minimum	Maximum	*p*
Does the farm perform stripping at the end of milking?	Yes, by hand	77	16.92	8.68	15.69	4.13	42.26	0.02
	Yes, by machine	106	15.23	7.77	13.50	2.94	36.43	
	No	115	17.45	7.29	16.86	3.88	40.00	
If stripping at the end of milking is performed, which animals are included?	All cows	62	14.93	7.60	13.37	2.94	42.26	0.03
	Some cows	131	16.37	8.43	15.06	4.13	40.00	
	It's not being done	96	17.56	7.23	16.90	3.88	34.63	
Is a milking order maintained (cows with increased cell counts, treated cows or cows known to have chronic udder diseases are milked last)?	Yes	70	14.90	7.83	13.62	2.94	40.00	0.03
	No	227	17.02	7.86	15.91	3.88	42.26	
Are the cows restrained at the feeding place after milking?	Yes	184	15.33	7.54	13.68	4.13	40.00	0.001
	No	112	18.27	8.07	17.37	2.94	42.26	
Are cows given fresh feed after milking?	Yes	266	16.08	7.70	15.26	2.94	40.00	0.017
	No	33	19.80	8.60	17.79	4.52	42.26	
Is teat dipping carried out after milking?	No	91	17.87	8.73	15.97	3.88	42.26	0.003
	It is done immediately after milking	192	15.45	7.15	14.61	2.94	40.00	
	Not done immediately after milking	14	22.13	7.97	18.80	6.67	33.22	
Is a bacteriological quarter milk test (milk sample and culture) performed before drying off?	Always	100	15.13	6.79	13.99	4.33	35.13	0.0004
	Only for suspicious animals	270	18.93	8.43	18.04	2.94	48.55	
	Never	40	18.37	8.26	17.69	5.69	37.17	
Is teat sealer used for drying off?	Never	178	18.37	8.58	17.42	3.88	48.55	0.048
	Only in individual cases	117	18.75	7.95	18.31	2.94	42.26	
	Yes, all the time	116	16.46	7.64	15.85	4.33	40	

Drying‐off by abrupt cessation of milking at the start of the dry period was associated with high milk yields (*p* < 0.05). Additionally, any changes in the drying‐off process over the previous decade negatively impacted milk yield (*p* < 0.05). Farms utilizing teat sealants during the dry period, rather than partial or inconsistent use, showed statistically significant higher milk yields (*p* < 0.05). In the farms included in this study, those with lower milk yield were found to apply preventive ketosis treatment to all cows, whereas farms that did not apply ketosis treatment or applied it only to a limited number of cows showed a considerably higher average milk yield (*p* < 0.05).

A positive correlation was recorded between the percentage of milk records exceeding 200,000/mL and the percent of cows exceeding the same threshold on three or more times during the observation period. In some farms, cows with high cell content or cows undergoing treatment or cows known to have chronic udder disease are milked last in the milking order. Lower amounts of SCC were detected in the milk of farms where cows with this condition were milked last, and this result was found to be statistically significant (*p* < 0.05). Having a separate feeding area for cows after milking was also associated with lower SCC. Statistically, keeping cows in feeding areas postmilking lowered SCC, as did providing fresh feed immediately following milking. Bacteriological cultures taken from all cows before drying off resulted in lower SCC compared to farms where this was done more seldom or not at all. The other factor was the use of teat sealants, which showed that farms solely using teat sealants without antibiotics showed lower SCC compared to farms that combined antibiotic‐sealant treatments (Table 4).

**TABLE 4:** Farm management practices shown in relation to the percentage of cows with SCC > 200,000/mL on three or more occasions during the observation period.

		Percentage of cows exceeding three times the SCC of 200,000/mL during the observation period						
Question	Category	Valid *N*	Mean	Standard deviation	Median	Minimum	Maximum	*p*
Are the cows restrained at the feeding place after milking?	Yes	184	14.68	9.73	12.96	0.00	48.57	0.012
	No	112	17.12	9.57	15.59	0.00	47.22	
Is teat dipping carried out after milking?	No	91	17.32	10.55	15.00	0.00	48.57	0.007
	It is done immediately after milking	192	14.42	9.04	13.51	0.00	47.62	
	Not done immediately after milking	14	21.72	10.26	22.79	3.92	37.74	
Is a bacteriological quarter milk test (milk sample and culture) performed before drying off?	Always	100	14.17	8.19	14.08	0.00	47.62	0.004
	Only in suspicious animals	270	18.15	10.21	16.43	0.00	50.59	
	Never	40	16.73	9.27	16.03	1.18	39.22	
Is teat sealer used for drying off?	Never	178	17.47	9.72	16.38	0.00	50.59	0.015
	Only in individual cases	117	18.39	10.29	16.67	0.00	48.57	
	Yes, always	116	14.97	9.09	13.92	0.00	42.31	

A positive association existed between the herd's percentage of milk records with SCC > 200,000/mL during the observation period and the percentage of cows exceeding this threshold three times within the same period. Table 3 presents the methods identified as effective to decrease farms' percentage of milk records with SCC > 200,000/mL during the study period.

A positive correlation was observed between the percentage of cows exceeding the SCC threshold of 200,000/mL on three or more occasions during the observation period and udder cleanliness graded at Level 2. The farm management practices that influenced the percentage of cows surpassing this SCC threshold are summarized in Table 4.

## 4 Discussion

Mastitis is an inflammation of the udder tissues that results from physical damage, chemical irritation, or infection. From an economic point of view, mastitis remains one of the costliest diseases in the dairy industry globally. The etiology of mastitis remains complex and not fully understood (Ashraf and Imran 2020). Hence, effective programs to control mastitis should focus on five areas of interest, including udder health, milking procedures, clinical mastitis treatment, dry cow management, and other management practices aimed at reducing the prevalence of mastitis and controlling all mastitis pathogens (Cobirka et al. 2020). The study explores how management practices in herds are associated with milk yield and mastitis prevalence in dairy farms in Austria.

One of the major economic effects of mastitis concerns a reduction in the volume of milk production (Jamali et al. 2018). The results show that udder dirtiness indices affect milk production. Grade 1 cleanliness is associated with higher yields, whereas Grades 3 and 4, which indicate high contamination levels, correlate with lower yields (Mıtev et al. 2012). This finding aligns with studies suggesting that inadequate udder hygiene is a risk factor for low milk yield (Fesseha et al. 2021). These findings highlight the importance of maintaining proper udder hygiene as part of herd management practices to optimize milk yield and reduce the economic losses associated with mastitis.

Results for udder cleanliness scores also explain the low average milk yield in farms, which is likely due to poor practices for cleaning the udder. In milking, the automatic cluster removal affects both milk yield and milking time (Magliaro and Kensinger 2005). In this regard, this research has indicated that application of automatic cluster removal positively reflected in average yield on farms. Farms that practiced postmilking teat dipping had a significantly higher average milk yield (*p* < 0.05) and fewer milk records with SCC exceeding 200,000/mL in the herd. Farms that performed milking followed by immediate teat dipping recorded a lesser number of cows with SCC over 200,000/mL more than three times during the study period (*p* < 0.05) (Table 4). The disinfection of the teat after milking is one of the most effective milking management practices for preventing mastitis (Cheng and Han 2020). The lack of teat dipping is a risk factor for mastitis (Mbindyo et al. 2020). Postmilking udder immersion, especially when premilking teat dipping is not done, is beneficial for mastitis prevention (Williamson and Lacy‐Hulbert 2013). Overall, these findings emphasize that appropriate milking hygiene practices—particularly the use of automatic cluster removal and postmilking teat dipping—play a crucial role in improving milk yield and reducing the incidence of mastitis within dairy herds.

Our findings underscore that strict adherence to proper milking techniques (machine stripping and sequencing) and postmilking protocols (restraint, immediate dipping, and fresh feed) are crucial for minimizing the proportion of high SCC milk records, along with a proactive approach to udder health during the dry period (Table 3). Milking order had also significant effects; farms that implemented a milking order reported higher yields than those without such an order (Table 3). Specifically, milking cows with SCC exceeding 200,000/mL or chronic mastitis after the healthy cows lowered SCC across the herd. This practice helps prevent the spread of infection and is shown to improve milk quality.

The results indicate that sound management practices—including restraining cows at the feeding place after milking (to keep them standing), immediate postmilking teat dipping, routine predrying‐off bacteriological quarter milk testing, and the consistent use of an internal teat sealant for drying off—significantly reduce the percentage of cows in the herd with a high SCC (Table 4).

A statistically significant relationship (*p* < 0.05) was identified between the milking methods (healthy cows were milked first, followed by cows with high SCC, those under treatment, or those with chronic mastitis) and the average milk yield among different milking practices on the farms. The proportion of milk records with SCC exceeding 200,000/mL was lower in farms using teat stripping, indicating its effectiveness in reducing SCC (*p* < 0.05). Although the overall milk average was lowest in farms applying teat stripping to all cows, it was highest in those applying it selectively to certain cows. Additionally, farms implementing teat stripping showed a low percentage of records where herd SCC exceeded 200,000/mL. Among milking methods, farms using teat stripping for cows with high SCC (Table 3), those undergoing treatment, or cows with chronic udder disease had significantly higher milk averages and lower SCC (*p* < 0.05). Studies on mastitis risk factors report that cows with mastitis are often not milked last (Mbindyo et al. 2020; Silva et al. 2021). Failing to milk sick animals last contributes to the spread of mastitis within the herd (Mbindyo et al. 2020). Our results highlight the importance of proper milking order and selective application of teat stripping as effective management strategies to improve udder health and maintain optimal milk yield in dairy herds.

A significant relationship was identified between various dry period management practices on farms and average milk yield. Similarly, a correlation was observed between SCC and dry period management methods. Farms that routinely performed bacteriological culture analysis on cows before drying had a lower percentage of milk records with SCC exceeding 200,000/mL and a lower proportion of cows with SCC surpassing this threshold more than three times, compared to farms where only suspicious animals were analyzed or no analysis was conducted.

The most statistically significant dry period management method for optimizing milk yield was the sudden cessation of milking (*p* < 0.05). Conversely, any changes to drying‐off practices within the past 10 years were found to have a statistically negative effect on milk yield (*p* < 0.05). Additionally, the use of teat sealant was significantly associated with an increase in milk yield, lower SCC, a reduced percentage of milk records SCC exceeding 200,000/mL, and fewer cows exceeding this SCC threshold more than three times, compared to farms that did not use teat sealants or used them selectively during the drying period (*p* < 0.05). It has been reported that the use of teat plugs during the dry period effectively prevents clinical mastitis (Berry and Hillerton 2002; Huxley et al. 2002). This study indicates that SCCs were higher in farms where a combination of teat sealants and antibiotics was used (*p* < 0.05). It is also possible that farms adopted both practices as a response to already elevated SCC levels.

Ketosis typically occurs in early lactation among high‐yielding cows the moment the energy balance becomes negative, further resulting in a serious decrease of milk production (Atalay 2019; King 1979). It was found that cows in the majority of the studied farms received preventive treatment for ketosis. However, in instances when no treatment against ketosis was applied, or when applied only to a few selected cows, milk production was considerably higher compared to farms with generalized treatment against ketosis (*p* < 0.05). A direct relationship exists between milk yield and energy requirements; thus, increased nutritional needs are one of the most crucial issues in the milking phase (Wang et al. 2021). These findings suggest that generalized preventive treatment for ketosis may not always be beneficial and could reflect differences in herd management or nutritional balance, emphasizing the importance of targeted prevention strategies based on metabolic risk assessment.

Providing each cow with access to a feed after milking was shown to effectively increase average milk yield and reduce the percentage of milk records with SCC exceeding 200,000/mL in the herd (*p* < 0.05). Additionally, the practice of keeping cows in the feeding area after milking significantly reduced both the herd percentage of SCC exceeding 200,000/mL and the number of cows that exceeded this SCC threshold more than three times. Offering fresh feed after milking was also effective at reducing SCC and the percentage of high SCC milk records present within the herd (*p* < 0.05). These findings again confirm the need for adequate nutrition right from the beginning of lactation to obtain optimal milk yield.

The strength of the study is that a larger data set on milk yield among others from approximately 400 dairy farms in Austria was collected and information on herd management was collected by surveys and statistically analyzed. In contrast, the weakness of the study is that not all participants answered all survey questions. Some survey questions were not fully answered, and homogeneous distribution could not be achieved in certain questions.

In conclusion, this paper carried out a comprehensive analysis of milk yields and mastitis records in Austrian dairy farms, with particular emphasis on the impact of management practices, including milking methods, dry period management, nutrition, disease prevention strategies, and udder health in relation to mastitis outcomes. These results indicate that management practices with respect to the dry period, nutrition, and milking methods have a profound impact both on milk yield and mastitis indicators. Based on these results, it is recommended that farmers receive training on effective strategies to enhance milk yield and control mastitis.

## Funding

This work was supported by the Austrian Federal Ministry of Digital and Economic Affairs, the Lower Austria and Vienna in the Framework of COMET‐Competence Centers for Excellent Technologies, and the Bundesministerium für Klimaschutz, Umwelt, Energie, Mobilität, Innovation und Technologie (872039).

## Conflicts of Interest

The authors declare no conflicts of interest.

## Supporting information


**Data S1:** Supplementary Information.

**Data S2:** Supplementary Information.
