# Supplementary material for: Assessment of the Herd Management Effects on Mastitis Frequency in Austrian Dairy Farms
Source: Anim Sci J. 2026 Jan 26;97(1):e70155. doi: 10.1111/asj.70155 (PMC12883582; doi:10.1111/asj.70155)
Supplement: Supplementary file 1 — Data S1: Supplementary Information. [file ASJ-97-e70155-s001.docx]

1. **The milking work is carried out ...**
   1. From a person
   2. By several people at the same time
   3. By several people alternately
2. **How long is the average duration of the milking work per milking time in minutes?**
3. **Are gloves worn during milking?**
   1. Yes, disposable gloves
   2. Yes, reusable gloves
   3. No
4. **How is the udder and teat cleaned?**
   1. Wet (e.g. with water) - without drying the udder before attaching
   2. Wet (e.g. with water) - udder is dried before attachment
   3. Moist (e.g. with foam or udder cloths)
   4. Dry (e.g. with paper or wood shavings)
   5. No cleaning
5. **Udder and teat cleaning are done with:**
   1. Udder flap (used several times)
   2. Disposable textile wipes
   3. Disposable paper
   4. Soap foam
   5. Udder wool
   6. Straw
6. **Is a disinfectant solution (e.g. iodine) used to clean the udder and teats?**
   1. Yes
   2. No
7. **Is milking pre-milked?**
   1. Yes, with examination of the foremilk in the pre-milk cup
   2. Yes, with examination of the foremilk by milking on the palm
   3. Yes, with foremilk testing by milking on the floor
   4. Yes, but without checking the foremilk for changes
   5. No
8. **Is there an automatic preparation system?**
   1. Yes
   2. Yes, but not used
   3. No
9. **Is there an automatic cluster removal system?**
   1. Yes
   2. Yes, but not used
   3. No
10. **Is there an automatic switch-off?**
    1. **Yes**
    2. Yes, but not used
    3. No
11. **Is there milking afterwards?**
    1. Yes, by machine
    2. Yes, by hand
    3. No
12. **Which animals are milked afterwards**
    1. All cows
    2. Single cows
    3. There is no subsequent milking
13. **Is an intermediate cluster disinfection carried out?**
    1. Yes, in all animals
    2. Yes, only for suspicious animals
    3. No
14. **How is intermediate disinfection carried out?**
    1. Dive
    2. Spray
    3. Automatically
15. **What is used for intermediate disinfection?**
    1. peracetic acid
    2. Chloramine-T
    3. Other (please specify)
16. **Is a milking sequence observed (cows with an increased cell content, treated cows or cows known to have chronic udder disease are milked at the end)?**
    1. Yes
    2. No
17. **Is there a feeding place for each cow after milking?**
    1. Yes
    2. No
18. **Are the cows restrained at the feeding place after milking?**
    1. Yes
    2. No
19. **Are fresh feed offered to the cows after milking?**
    1. Yes
    2. No
20. **Is there teat dipping after milking?**
    1. Does not occur
    2. Takes place immediately after the milking cluster is removed
    3. Does not take place immediately after cluster removal
21. **Teat dipping is done by means of...**
    1. Dip cup
    2. Spray device
22. **Which preparation/agent is used for teat dipping?**
23. **How often are old dip/spray solutions changed?**
    1. Before each milking
    2. Daily
    3. Less often
24. **Is a bacteriological quarter milk test (milk sample and culture) carried out before drying off?**
    1. Regularly
    2. Only for suspicious animals
    3. Never
25. **What measures are taken if there are positive bacteriological results when drying off?**
    1. Therapy before drying off (other than antibiotic drying off)
    2. Only antibiotic dry-off
    3. Other (please specify)
26. **How does the drying off take place?**
    1. Abruptly (no more milking at all)
    2. Gradually
    3. Adjusted drying off - abruptly or gradually depending on the cow
27. **How many days before the target calving date are the cows dried off?**
    1. More than 60 days
    2. 56-60 days (2 months before calving date)
    3. 49-55 days
    4. 42-48 days
    5. 35-41 days
    6. Less than 35 days before calving
    7. Changing drying time
    8. Other (please specify)
28. **At what intervals are the udders checked during the dry period (including visual inspection)?**
    1. Daily
    2. 2 times a week
    3. Weekly
    4. Every 2 weeks
    5. Monthly
    6. Irregular
    7. Rarely
    8. Never
    9. Other (please specify)
29. **Have there been any changes in drying off in the last 10 years (procedure when drying off, BU, use of teat sealers, antibiotic drying off)?**
    1. Yes
    2. No
30. **Since which year have the conditions in the following areas been as reported in this survey? (If the changes are more than 10 years old, no information is required for the respective area.)**

Carrying out a BU before drying off

How to proceed drying off

Use of antibiotic Other (please specify) dry cow

Use of teat sealers

Udder check the drying off

1. **Is drying off with antibiotics?**
   1. Yes, with all cows
   2. Only in animals with positive bacteriological results
   3. Only for other suspicious animals (e.g. swollen udder, milk changes, scarf test positive, etc.)
   4. No (no use of antibiotic drying agents)
2. **If drying off with antibiotics is only for suspect animals, what are the criteria for use?**
   1. Increased cell count/scum test positive
   2. Mastitis during lactation
   3. Swollen udder quarter
   4. Milk changes
   5. LKV data
3. **Are teat sealers used for drying off?**
   1. Yes, regularly
   2. Only in individual cases
   3. No never
4. **How are teat sealers used for drying off?**
   1. In combination with an antibiotic drying agent
   2. Alone (not in combination with an antibiotic dry-off)
   3. Varies from cow to cow - both in combination with antibiotic drying off and alone used
5. **Is preventive ketosis treatment being carried out?**
   1. Yes, with all cows
   2. Only for selected cows
   3. No
6. **Are the berths mostly clean? (A clean berth is defined as no more than spot-on soaking of the berth; max. one drop of feces)**
   1. Yes (more than 2/3 of the berths are clean)
   2. No (more than 1/3 are dirty)
7. **Is the herd clean? (Assessment: Consider the entire hindquarters on one side of the body, from the coronet to the pelvis, i.e. including the underfoot: soiled if there are (continuous) deposits of excrement longer than the forearm length)**
   1. Yes, more than 2/3 of the animals are clean
   2. No, more than 1/3 of the animals have (continuous) droppings of more than forearm length on the hindquarters
8. **How many animals have hock injuries or swelling? (Assessment of one side in the milking parlor (alternatively, the outside of one leg and the inside of the other leg can be assessed) % please determine by comparing the number of animals with injuries compared to the total number of animals assessed)**
9. **Udder contamination in the herd? (please enter % per grade, total should be 100%)**

Grade 1

Grade 2

Grade 3

Grade 4

1. **Are the teats (after cleaning) clean before attaching the cluster?**
   1. Yes
   2. No
2. **How many cows lie down in the cubicle/lying area immediately after milking?**
   1. None (0%)
   2. No more than 10%
   3. More than 10%
   4. No assessment because AMS
